# Structure-Guided Approach to Discover Tuberosin as a Potent Activator of Pyruvate Kinase M2, Targeting Cancer Therapy

**DOI:** 10.3390/ijms232113172

**Published:** 2022-10-29

**Authors:** Mohd Adnan, Anas Shamsi, Abdelbaset Mohamed Elasbali, Arif Jamal Siddiqui, Mitesh Patel, Nawaf Alshammari, Salem Hussain Alharethi, Hassan H. Alhassan, Fevzi Bardakci, Md. Imtaiyaz Hassan

**Affiliations:** 1 Department of Biology, College of Science, University of Ha’il, Ha’il P.O. Box 2440, Saudi Arabia; 2 Molecular Diagnostics and Personalized Therapeutics Unit, University of Ha’il, Ha’il P.O. Box 2440, Saudi Arabia; 3 Centre for Interdisciplinary Research in Basic Sciences, Jamia Millia Islamia, Jamia Nagar, New Delhi 110025, India; 4 Department of Clinical Laboratory Science, College of Applied Medical Sciences-Qurayyat, Jouf University, Sakaka P.O. Box 72388, Saudi Arabia; 5 Department of Biotechnology, Parul Institute of Applied Sciences and Centre of Research for Development, Parul University, Vadodara 391760, India; 6 Department of Biological Science, College of Arts and Science, Najran University, Najran P.O. Box 11001, Saudi Arabia; 7 Department of Clinical Laboratory Science, College of Applied Medical Sciences-Sakaka, Jouf University, Sakaka P.O. Box 72388, Saudi Arabia

**Keywords:** pyruvate kinase M2, phytoconstituents, tuberosin, virtual screening, molecular dynamics simulations, principal component analysis, kinase activator, drug discovery

## Abstract

Metabolic reprogramming is a key attribute of cancer progression. An altered expression of pyruvate kinase M2 (PKM2), a phosphotyrosine-binding protein is observed in many human cancers. PKM2 plays a vital role in metabolic reprogramming, transcription and cell cycle progression and thus is deliberated as an attractive target in anticancer drug development. The expression of PKM2 is essential for aerobic glycolysis and cell proliferation, especially in cancer cells, facilitating selective targeting of PKM2 in cell metabolism for cancer therapeutics. We have screened a virtual library of phytochemicals from the IMPPAT (Indian Medicinal Plants, Phytochemistry and Therapeutics) database of Indian medicinal plants to identify potential activators of PKM2. The initial screening was carried out for the physicochemical properties of the compounds, and then structure-based molecular docking was performed to select compounds based on their binding affinity towards PKM2. Subsequently, the ADMET (absorption, distribution, metabolism, excretion and toxicity) properties, PAINS (Pan-assay interference compounds) patterns, and PASS evaluation were carried out to find more potent hits against PKM2. Here, Tuberosin was identified from the screening process bearing appreciable binding affinity toward the PKM2-binding pocket and showed a worthy set of drug-like properties. Finally, molecular dynamics simulation for 100 ns was performed, which showed decent stability of the protein-ligand complex and relatival conformational dynamics throughout the trajectory. The study suggests that modulating PKM2 with natural compounds is an attractive approach in treating human malignancy after required validation.

## 1. Introduction

The difference in metabolism is one of the key attributes distinguished between normal and cancerous cells [1]. Unlike normal cells, cancer cells metabolize glucose by aerobic glycolysis following the Warburg effect phenomenon [2]. Pyruvate kinase M2 (PKM2) is a phosphotyrosine-binding protein that plays a vital role in cancer progression by both metabolic and non-metabolic pathways [3]. Its altered expression has been observed in many human cancers and had a crucial role in metabolic reprogramming, transcription and cell cycle progression [4,5]. It is involved in the resolution of tumor growth by triggering gene expression, which is involved in migration, cell proliferation, and apoptosis [6]. 

PKM2 was found in several cancers and promoted metastasis and proliferation of cancer cells [7]. Due to its altered expression in cancer cells and critical role in aerobic glycolysis and cell proliferation, PKM2 is considered an attractive target in anticancer drug development [5]. It contributes an essential function for tumor metabolism, thus serving as a promising target for anticancer therapeutics. Therapeutic strategies targeting PKM2 are believed to be an excellent option for repressing cancer [5,8]. 

Several activators, inhibitors, and hormones block cell proliferation by targeting PKM2 [9,10,11,12]. Multiple PKM2 activators and inhibitors are in pre-clinical and clinical studies, which suggests their promising anticancer potential [8]. The relationship of PKM2 with many external factors influences the metabolic actions of tumor cells in various ways [8]. Studies have identified that shRNA (short hairpin RNA) and miRNA (microRNA) obstruct the PKM2 expression, which initiates the cell death of tumor cells, reduces metabolic activity, and decreases tumorigenesis [13]. shRNA hinders the expression of PKM2, the sensitivity of cancer cells to certain agents or drugs, including docetaxel and cisplatin, that induces the cell death of cancer tissues and decreases tumorigenesis [14,15].

Drug development strategies targeting PKM2 are predominantly achieved by activating/down-regulating PKM2, inhibiting its enzymatic activity, and stimulating dimer activity [11,16]. The binding prototype of the PKM2 activators has been explained by various structural studies and high-throughput screening, which might help develop specific leads [11,12,17,18,19]. Structure-based computational approaches, such as molecular docking and molecular dynamics (MD) simulations, can assist in the discovery of novel compounds targeting PKM2 for therapeutic applications [20,21,22]. These approaches are predominantly implemented in the modern drug discovery pipeline to discover potential leads from different compounds libraries, such as PubChem [23], DrugBank [24], ZINC database [25], IMPPAT (Indian Medicinal Plants, Phytochemistry And Therapeutics) database [26], etc. 

Here we employed a virtual screening approach to find potential binding partners of PKM2. This study screens a library of phytoconstituents in the IMPPAT database of compounds from Indian medicinal plants. After collecting compounds based on their physicochemical properties, molecular docking studies were performed on the selected compounds for their binding affinities. The high-affinity compounds were further subjected to interaction analysis followed by their absorption, distribution, metabolism, excretion and toxicity (ADMET) and PASS evaluation. We identified Tuberosin bearing appreciable binding affinity toward the PKM2 and a worthy set of drug-like properties. Finally, all-atom MD simulation studies were performed for 100 ns to evaluate the structural dynamics and time-evolution of PKM2-Tuberosin interactions and their stability. These data demonstrate that therapeutic targeting of PKM2 with natural compounds is suitable for targeting cancer metabolism for therapeutic management. 

## 2. Results and Discussion 

### 2.1. Molecular Docking-Based Virtual Screening

Molecular docking-based virtual screening is a computational approach used in identifying potential leads against predefined targets [22,27]. It was used to find the compounds with appreciable binding affinities and specific interactions toward PKM2. The docking output of the RO5 filtered library of 5875 compounds resulted in identifying several compounds with an appreciable affinity towards PKM2. The top 10 selected showed an affinity score from −8.9 kcal/mol to −10.0 kcal/mol (Table 1). The docking result showed that all the chosen hits showed higher affinity than the reference compound. The results suggested that the selected phytoconstituents have appreciable binding efficiency with PKM2, which might be used in developing a potential binding partner of PKM2 for therapeutic development. 

### 2.2. ADMET Properties

The ADMET properties, along with the PAINS patterns of all the selected compounds, were predicted through the pkCSM and SwissADME web servers. The ADMET properties of all the compounds are given in Table 2. The results showed that four compounds have good ADMET properties with no PAINS patterns. Out of 10 compounds, 6 had AMET toxicity, which needed to be excluded for further analysis. ADMET properties revealed that four compounds (Lupinisolone C, Gummadiol, 2,3-Dehydrokievitone, and Tuberosin) share a similar class of ADMET properties without any toxic patterns. Examining the AMDET properties indicated that these four compounds might potentially be a potent and safe lead for anticancer drug development. 

### 2.3. PASS Evaluation

Phytoconstituents retain numerous biological properties, possibly resulting in synergistic or antagonistic effects [28,29]. Finding safe and effective lead molecules for drug development needs an assessment of the biological properties of the compound under study. PASS analysis was performed to explore the plausible biological properties of the selected compounds. Here, the selected compounds were evaluated based on their multiple biological properties and confidence level (Table 3). The results showed that two compounds, Lupinisolone C and Tuberosin, possess antioxidant, anticarcinogenic, antineoplastic, and kinase binding potential, with considerable Pa values ranging from 0.792 to 0.908. However, Gummadiol and 2,3-dehydrokievitone also exhibit similar properties but with lower Pa values; hence were eliminated from the study for further study. The PASS evaluation and the molecular docking study and ADMET properties suggested that Lupinisolone C and Tuberosin have great potential to explore in anticancer drug discovery pipeline targeting PKM2 for therapeutic development.

### 2.4. Interaction Analysis

**Figure 1:**
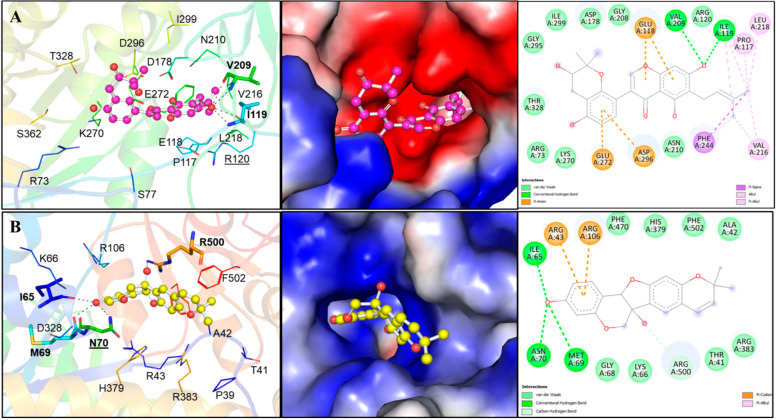
Structural representation of PKM2 in complexed with Lupinisolone C and Tuberosin. (**A**) Molecular interactions of Lupinisolone C with PKM2. Protein is shown in rainbow ribbon representation, where the interacting residues and hydrogen bonding are shown in lines and sticks, respectively. The middle panel shows the charged surface illustration of the PKM2 binding pocket occupied by Lupinisolone C. The right panel shows the 2D structural representation of PKM2 residues and their interaction with Lupinisolone C (**B**) Molecular interactions of Tuberosin with PKM2. The left panels show cartoon representations of the interactions. Protein is shown in rainbow ribbon representation, where the interacting residues and hydrogen bonding are shown in lines and sticks, respectively. The middle panels show the charged surface illustration of the PKM2 binding pocket occupied by Tuberosin. The right panel shows 2D structural representation of PKM2 residues and their interaction with Tuberosin.

Two compounds, Lupinisolone C and Tuberosin, were selected for interaction analysis and it found that both compounds interact with several residues of PKM2 and share a common interaction pattern. The detailed binding pattern of Lupinisolone C and Tuberosin with PKM2 is illustrated in Figure 1. The interaction analysis of Lupinisolone C suggested that it was stabilized by two H-bonds and multiple hydrophobic interactions (Figure 1A). Lupinisolone C’s interaction was near an ATM-binding site, i.e., Arg120 [12]. At the same time, Tuberosin was found to interact with the crucial residues of the PKM2 binding pocket, including the Serine binding site ‘Asn70’ [11] (Figure 1B). This binding site is crucial for the PKM2 activity as Serine act as a natural ligand and allosteric activator of PKM2 [11].

Tuberosin was fitted within the binding pocket of PKM2, having various close interactions. The binding of Tuberosin with PKM2 was stabilized by several interactions, including four H-bonds and a few hydrophobic interactions. Detailed interaction analysis showed that four H-bonds stabilized the PKM2-Tuberosin complex with Ile65, Met69, Asn70, and Arg500, two π-cation binds with Arg43 and Arg106, along with eight van der Waals interactions (Figure 1B, right panel). The stable binding of Tuberosin to the Serine binding site might be vital to activate the kinase activity of PKM2 and can raise Tuberosin as a “competitive activator”. Consequently, it can be suggested that Tuberosin may enhance the catalytic activity of PKM2 and thus may be a potential lead in developing anticancer therapeutics.

### 2.5. Structural Deviations in PKM2

**Figure 2:**
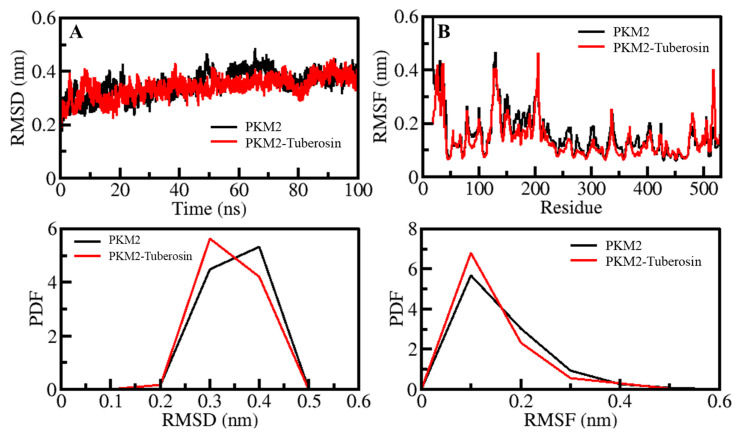
Dynamics of PKM2 and its binding with Tuberosin. (**A**) RMSD plot of PKM2 before and after Tuberosin binding. (**B**) RMSF plot of PKM2 and its complex with Tuberosin. Lower panels show the distribution of parameters as PDF.

Before analyses of MD trajectories for the time evolution of different parameters, total potential energy and time were checked to know whether the systems had reached equilibrium. These parameters can affect the results of the MD calculations. The results showed that both the simulated systems reached equilibrium and 100 ns of time (Figure 2). These trajectories were compacted and subjected to various systematic and structural parameters for further analysis.

The structural movements in a protein are essential for its functional activity inside the living system [30]. The analysis of RMSD has been convenient for examining the structure deviation in proteins and protein-ligand complexes [31,32]. The structural deviations of the PKM2 and PKM2-Tuberosin complexes were examined within the solvent environment to check their stability and movements during the simulation. The RMSD values of the backbone of PKM2 and its docked complex with Tuberosin were recorded to examine their structural deviations, which remained stable during the entire simulation (Figure 2A, upper panel). The average values of RMSD for PKM2 and PKM2-Tuberosin complex were 0.35 nm and 0.34 nm with a maximum of 0.48 nm and 0.45 nm at 65 ns and 90 ns, respectively. A random but negligible fluctuation in the RMSD pattern was seen 30 ns in the case of both systems of PKM2, possibly due to their initial adjustment. Overall, the distribution of the RMSD pattern did not show any substantial shifts, which suggested the stability of PKM2 with a palpable strength of ligand binding during the simulation. The distribution of RMSD values as a probability distribution function (PDF) also suggested no significant change in the PKM2 dynamics after the Tuberosin binding (Figure 2A, lower panel).

Studying RMSF indicates the flexibility of each residue in a protein [33]. To examine the residual vibrations in PKM2 before and after Tuberosin binding, the RMSF values of each residue were recorded (Figure 2B). The average fluctuations in PKM2 and PKM2-Tuberosin complex were 0.16 nm and 0.14 nm during the simulation. The fluctuations were observed to be stable and minimized upon Tuberosin binding. The graph suggested a remarkable constancy of the docked complex of PKM2 and Tuberosin interaction. The major fluctuations can be observed in the loop and coils, where residues are not involved in the ligand binding. A little decreased fluctuation was seen in the PKM2 residues after the Tuberosin binding signified reduced dynamics in the ligand-binding pocket of PKM2 (Figure 2B, upper panel). The PDF of RMSF distribution also depicted decreased fluctuation in PKM2 after Tuberosin binding (Figure 2B, lower panel).

**Figure 3:**
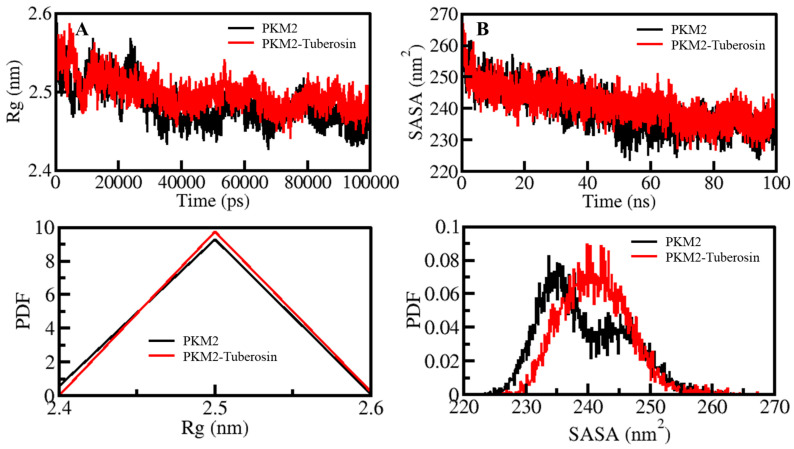
Structural compactness and folding of PKM2 after Tuberosin binding. (**A**) The *R_g_* distribution as a function of time. (**B**) SASA plot of PKM2 as a function of time before and after Tuberosin binding. The lower panels show the distribution of parameters as a PDF.

The compactness of protein is another measure of computing the stability of protein molecules [34]. In MD simulations, the compactness measure is characterized as *R_g_* [35]. It is a useful parameter directly associated with the tertiary structure of a protein and has been widely employed in examining the compactness of a protein structure [36,37]. The compactness of PKM2 before and after Tuberosin binding was assessed by investigating the time evolution of the *R_g_* values. The average values of *R_g_* for PKM2 and PKM2-Tuberosin were 2.48 nm and 2.49 nm, respectively (Figure 3A). As suggested by the *R_g_* plot, the compactness of both systems persisted throughout the simulation without any significant shift (Figure 3A, upper panel). However, a minor increase in the *R_g_* values of PKM2 after Tuberosin binding can be in agreement with the assumption that there was occupancy of intramolecular space in PKM2 by Tuberosin, which caused an increment of *R_g_*. The PDF distribution of the *Rg* values also suggested the proper compactness of PKM2 in the presence of Tuberosin (Figure 3A, lower panel).

SASA of a protein molecule is the surface area that is accessible to its neighbouring solvent. The SASA analysis has been widely utilized in examining the folding/unfolding of proteins, thus, their structural stability during the simulation [38]. The effect of Tuberosin binding on the folding behaviour of PKM2 was examined by calculating SASA values which showed no manor peaks during the simulation. The average SASA values for PKM2 and PKM2-Tuberosin were 239.34 nm^2^ and 241.23 nm^2^, respectively. The graph of the time evolution of SASA values suggested that PKM2 was a stable presence of Tuberosin (Figure 3B, upper panel). The SASA values distribution showed a similar pattern in both systems. The PDF distribution of the SASA values suggested a minor increase in the SASA values of the PKM2-Tuberosin docked complex (Figure 3B, lower panel).

### 2.6. Dynamics of Hydrogen Bonds

**Figure 4:**
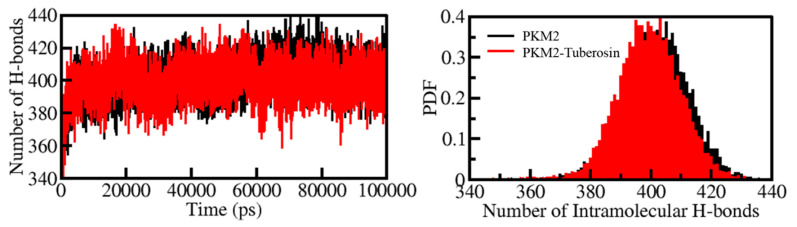
The dynamics of intramolecular H-bonds in PKM2 (left panel). The probability distribution of the intramolecular H-bonds in PKM2 (right panel).

H-bonds play a vital role in the stability and integrity of protein structures [39]. Examining the time evolution of formation and breakdown of H-bonds during the simulation time is useful for assessing the structural integrity and stability of proteins and protein-ligand complexes [40]. The time evolution of H-bonds was calculated and plotted to examine the consistency of intramolecular bonds within PKM2. The average number of H-bonds formed intramolecularly within PKM2 before and after Tuberosin binding were 402 and 399, respectively (Figure 4, left panel). The results suggested no significant change in the number of H-Bonds within PKM2 when complexed with Tuberosin. A negligible fall in the number of H-bonds can be correlated with the occupancy of intramolecular space in PKM2 by Tuberosin. The calculated PDF suggested a fair constancy in intramolecular H-bonds in PKM2 before and after Tuberosin (Figure 4, right panel).

**Figure 5:**
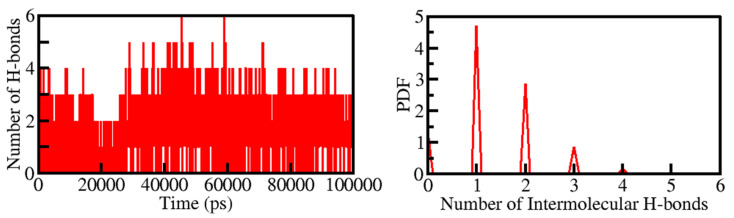
Time evolution of intermolecular H-bonds formed within 0.35 nm between Tuberosin and PKM2 (Left panel). The right panel shows the PDF of the intermolecular H-bonds between Tuberosin and PKM2.

Intermolecular H-bonding formed within the PKM2-Tuberosin docked complex makes the protein-ligand complex stable. The presence of four H-bonds maintained the PKM2-Cital docked complex. Hence their time-evolution was examined during the simulation (Figure 5). The plot indicated that an average of three H-bonds was formed between Tuberosin and PKM2, which were quite stable during the simulation. The result suggested that Tuberosin forms up to five H-bonds fluctuated at several places, but up to three H-bonds were maintained throughout the simulation. The distribution plot also suggested that three H-bonds were formed within the PKM2-Tuberosin docked complex with the highest PDF (Figure 5, right panel).

### 2.7. PCA and FELs Analyses

**Figure 6:**
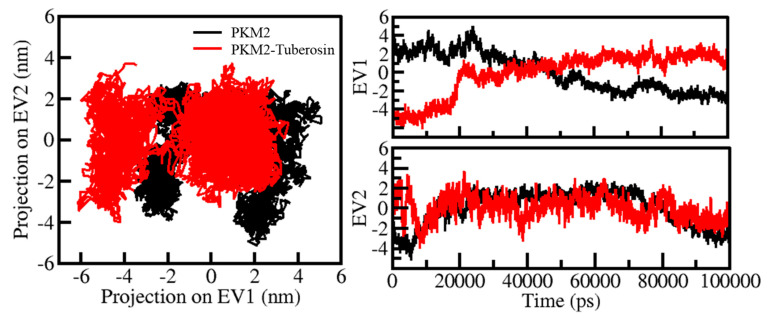
Conformational projections of PKM2 in PCA. 2D projections of conformational sampling of PKM2 and PKM2-Tuberosin (left panel). The time evolution of projections of trajectories on both EVs (right panel).

Protein conformations play a vital role in their biological function [41]. Exploring the structural conformations in proteins has been useful in examining their function and stability during the simulation [42]. PCA has been a useful approach to exploring the atomic motions in proteins making their conformational projections [43]. The PCA on all Cα atoms PKM2 was carried out on PC1 and PC2 phase space. The projection of PKM2 in apo and ligand-bound states was thrown on a two-dimensional subspace covered along two PCs, i.e., PC1 and PC2 (Figure 6). In Figure 6, the highly populated dense area showed a steady state of protein conformation. The recorded projection of PKM2 PCs contributed to a variance of −6 and 2 on PC1 and −4 and 4 on PC2 (Figure 6, left panel. At the same time, the contribution of the ligand-bound state of PKM2 PCs to the variance was −5 and 5 on PC1 and −5 and 2 on PC2 (Figure 6, right panel). The conformational behaviors of PKM2 before and after Tuberosin binding showed a noteworthy difference in their stable projections along the PC1 and PC2. The distribution of PKM2-Tuberosin projections was more concentrated than the free state of PKM2, suggesting the stability of the docked complex.

**Figure 7:**
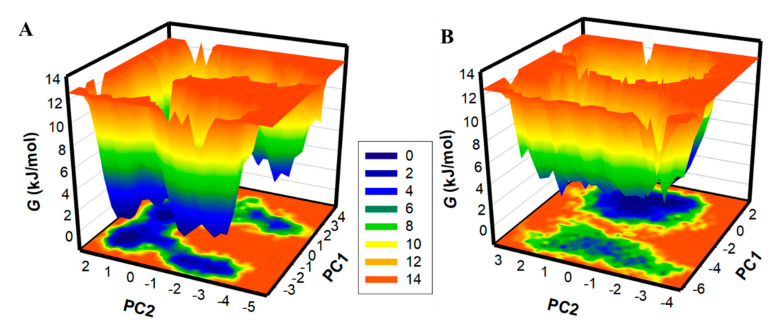
The FEL plots for (**A**) PKM2 and (**B**) PKM2-Tuberosin complex.

Generating FELs and analyzing them is useful for describing the ageing of the protein folding mechanism [44]. FELs have been utilized in the drug discovery process to examine the effect of ligand binding on protein folding and structure stability [27]. Here, FELs were generated to see the global minima and conformational landscape of PKM2 before and after Tuberosin binding (Figure 7). Deeper blue in the FELs indicated the protein conformational state with lower energy near the global minimum. The FEL plots indicated that PKM2 showed a single global minimum confined within a large basin (Figure 7A). The analysis suggested that the Tuberosin binding to PKM2 slightly disturbed the position of the phase but within a single stable global minimum (Figure 7B). The results suggested that the binding of Tuberosin to PKM2 was stable in the simulation course, which further supported Tuberosin as a potential binding partner of PKM2 for therapeutic development.

### 2.8. MMPBSA Binding Free Energy

The binding free energy of Tuberosin with PKM2 was estimated from the MD trajectory of a 10 ns stable region, i.e., between 40 to 50 ns. The analysis indicated that Tuberosin shows an appreciable binding affinity with PKM2, i.e., −92.84 ± 18.26 kJ/mol. The MMPBSA result supported the observation that Tuberosin binds to PKM2 with an appreciable binding affinity.

## 3. Materials and Methods

### 3.1. Web Resources and Computational Settings

The three-dimensional structure of PKM2 protein was taken from the Protein Data Bank (PDB ID: 6V76). All hetero atoms, including water and co-crystallized ligands, were deleted from the downloaded coordinates. The PKM2 structure was preprocessed for molecular docking-based virtual screening in the Swiss PDB Viewer tool [45] by restoring missing atoms and minimizing overall energy. The compound library from the IMPPAT database was downloaded that consisted of ~9000 small molecules, including phytoconstituents from Indian medicinal plants. All the compounds were filtered in such a way that they adhered to Lipinski’s rules [46] (i.e., molecular weight < 500, H-bond donors ≤ 5, H-bond acceptors ≤ 10, and logP < 5) and have three-dimensional coordinates optimized for molecular docking study where they left to 5875 in total. A comprehensive computational approach to drug discovery using different bioinformatics software, such as InstaDock [47], Discovery Studio Visualizer [48], PyMOL [49], GROMACS [50], QtGrace [51], etc., were employed for molecular docking, visualization, and simulation studies. 

### 3.2. Molecular Docking-Based Virtual Screening

Molecular docking-based virtual screening was performed to filter out the compounds based on their binding affinity towards PKM2. The docking was performed using InstaDock software using a blind search space for all the ligands. The grid was set to 96 Å, 75 Å, and 69 Å for X, Y, and Z coordinates. The grid centre was chosen at −15.342, −29.605, and −9.474 for the X, Y, and Z-axis, respectively. The grid was big enough to cover the entire protein so that each could move and find its promising binding sites. The grid spacing was fixed to 1 Å with default docking parameters. The docking score of each compound towards PKM2 was estimated and analyzed through InstaDock. The output of the screening process was filtered out based on the docking score. For further interaction analysis, all possible docked conformers of each ligand were split through the ‘Splitter’ program of InstaDock. 

### 3.3. ADMET Prediction

The filtered compounds from the docking results were subjected to filtering out based on their ADMET properties. The prediction of ADMET properties along with PAINS (Pan-assay interference compounds) evaluation were carried out using the pkCSM [52] and SwissADME [53]. Compounds with well ADMET properties were taken and then filtered for any PAINS patterns [54]. PAINS filter helps us to avoid compounds having specific patterns with a higher tendency to bind to multiple targets. The ADMET evaluation helps find compounds with drug-like physicochemical and pharmacokinetic properties, which reduces their chances of failure in clinical trials [55]. 

### 3.4. PASS Analysis 

PASS is the ‘prediction of activity spectra for biologically active substances’ where the biological activities of a chemical compound can be predicted through its chemical structure. PASS analysis through the PASS web server helps select compounds with desired biological properties [56]. PASS evaluation predicts possible biological properties for a compound based on structure-activity relationships. It provides a list of probable properties for a compound on the ratio of Pa (probability of being active) and Pi (probability of being inactive). A higher Pa value signifies a higher likelihood of the activeness of that compound with a particular property. The Pa value was set to the cut of >7 for higher statistical significance.

### 3.5. Interaction Analysis

The interaction analysis of the docked protein-ligand complexes was performed to explore various interactions formed during their binding. The binding poses and all possible interactions were explored through the PyMOL and Discovery Studio Visualizer [57]. The interactions formed within 3.5 Å within the protein-ligand complex were labelled in the PyMOL. The type of interactions and the participating residual and atomic coordinates were explored through Discovery Studio Visualizer. Here, only compounds with specific interactions towards the critical residues of PKM2, including the active and binding sites, were selected for further analyses. The binding of known PKM2 binding partners was referred to compare docking outputs. 

### 3.6. MD Simulations

It is important to analyze the stability of the protein-ligand complex in MD simulations. The all-atom MD simulation studies were carried out by the GROMACS version 5.1.2 [58]. The structural coordinates of the PKM2 and PKM2-Tuberosin complexes were prepared from the docking study for the starting point for our MD simulation setup. Protonate3D was used for protonation and to optimize hydrogen bonding. The GROMOS force field embedded in GROMACS was utilized for both simulations. The SPC solvent model was used for solvation purposes. The topology PKM2 was generated by the GROMACS, while the Tuberosin topology was generated through an external server named PRODRG [59]. The systems were neutralized using sufficient sodium and chloride ions at 0.15 M using the *gmx genion* module of the GROMACS. Equilibration of both systems was performed in two stages, i.e., NVT and NPT. In the NVT ensemble, the systems were heated gradually to 300 K for 1000 ps. At that time, in the NPT ensemble, the systems allowed the solvent molecules to relax for 1000 ps, removing any restraints from the systems. The pressure of 1 bar was preserved by the Berendsen Barostat method. The LINCS algorithm controlled the bond length between the protein-ligand complexes, whereas the SETTLE restrained water. Energy minimization was performed using the 1500 steps of the steepest descent algorithm. Finally, both the equilibrated systems were subjected to simulation for 100 ns maintaining supplied temperature and pressure for MD production. The time evolution data of MD trajectories were analyzed through the GROMACS package. 

### 3.7. Principal Component Analysis and Free Energy Landscapes 

Principal component analysis (PCA) is a convenient approach for exploring the conformational sampling of proteins [20]. The MD trajectories of PKM2 and PKM2-Tuberosin were examined to study their principal motions through PCA. The PCA was performed through the essential dynamics (ED) method by diagonalizing the first two eigenvectors (EVs) of the covariance matrix [60]. Further, free energy landscapes (FELs) for PKM2 and PKM2-Tuberosin complex were generated to examine their stability and folding dynamics. FELs were generated via a conformational sampling approach, which helps understand the conformational stability PKM2 and PKM2-Tuberosin complex during the simulation. 

### 3.8. MM-PBSA Calculations 

The binding of Tuberosin with PKM2 was further evaluated by calculating the binding affinity of the docked complex using the MM–PBSA approach [61]. The *g_mmpbsa* package of GROMACS was utilized to perform the MM–PBSA analysis [62]. The trajectory from a stable region, i.e., between 40 to 50 ns, was used for the calculation.

## 4. Conclusions

This study proposed Tuberosin as a potential lead for drug development targeting PKM2. The molecular docking approach explored the binding pattern of Tuberosin with PKM2. It was found that Tuberosin was docked well with PKM2 and showed appreciable binding affinity by forming the H-bonds with Ile65, Met69, Asn70, and Arg500. Apart from the H-bonds, Tuberosin maintained several Van der Waals interactions. Tuberosin possesses a set of drug-like properties and anticancer potential. Consequently, an all-atom MD simulation on PKM2 and PKM2-Tuberosin docked complex was carried out for 100 ns to determine their dynamics and stability. The MD analyses by exploring the time evolution of RMSD, RMSF, *R_g_*, SASA, and intra/intermolecular H-bonds suggested that PKM2 and PKM2-Tuberosin complex reached equilibrium by 40 ns and showed stability throughout the simulation trajectories. The PCA and FEL analyses examined the conformational movements and folding mechanism. It was found that the PKM2-Tuberosin complex was more stable than the free form of PKM2. In brief, this study could deliver an effective platform in developing potential leads of therapeutic potential against cancer, targeting PKM2. However, this work is based on multiple in-silico methods following state-of-the-art drug discovery approaches, which need further evaluation in experimental settings.

## Figures and Tables

**Table 1:** Selected hits, their source and binding affinities, and their ligand efficiency toward PKM2. Two compounds (PDB IDs: 07T and 1OX were taken as references for docking score comparison).

S. No.	Compound ID	Compound Name	Source (Plant)	Affinity (kcal/mol)	Ligand Efficiency
1.	CID:14237667	Lupinisolone C	*Lupinus albus*	−10.0	0.29
2.	CID:13846202	11-Hydroxytephrosin	*Amorpha fruticosa*	−9.4	0.28
3.	CID:12305449	Kanjone	*Pongamia pinnata*	−9.1	0.36
4.	CID:10308017	Gummadiol	*Gmelina arborea*	−9.1	0.30
5.	CID:108026-27-3	2,3-Dehydrokievitone	*Vigna radiata*	−9.0	0.32
6.	CID:104940	O-Methylsterigmatocystin	*Matricaria recutita*	−8.9	0.32
7.	CID:14630496	Tuberosin	*Pueraria lobata*	−8.9	0.32
8.	CID:125848	Alteichin	*Eichhornia crassipes*	−8.9	0.31
9.	CID:177032	Dihydrooroxylin A	*Glycyrrhiza glabra*	−8.9	0.37
10.	CID:14237662	Lupinisoflavone H	*Lupinus albus*	−8.9	0.26
11.	PDB ID: 07T	6-(3-aminobenzyl)-4-methyl-2-methylsulfinyl-4,6-dihydro-5H-thieno [2′,3′:4,5]pyrrolo[2,3-d]pyridazin-5-one		−7.4	0.27
12.	PDB ID: 1OX	2-(1H-benzimidazol-1-ylmethyl)-4H-pyrido[1,2-a]pyrimidin-4-one		−6.5	0.30

**Table 2:** ADMET properties of the selected compounds. GI, gastrointestinal; BBB, blood-brain barrier; CNS, central nervous system; CYP2D6, Cytochrome P450 2D6.

Compound	Absorption		Distribution	Metabolism	Excretion	Toxicity
	*GI* *Absorption*	*Water Solubility*	*BBB/CNS Permeation*	*CYP2D6* *Inhibitor*	*OCT2 Substrate*	*AMES*
Lupinisolone C	High	Poor	No	No	No	No
11-Hydroxytephrosin	High	Moderate	No	No	No	Yes
Kanjone	High	Moderate	Yes	No	No	Yes
Gummadiol	High	Soluble	No	No	No	No
2,3-Dehydrokievitone	High	Poor	No	No	No	No
O-Methylsterigmatocystin	High	Moderate	Yes	No	No	Yes
Tuberosin	High	Moderate	Yes	No	No	No
Alteichin	High	Soluble	No	No	No	Yes
Dihydrooroxylin A	High	Moderate	Yes	No	No	Yes
Lupinisoflavone H	High	Poor	No	No	No	Yes

**Table 3:** Biological and structural properties of the selected compounds predicted through the PASS webserver Pa (probability of being active) and Pi (probability of being inactive) values signify the likelihood of activeness of a compound with a particular property.

S. No.	Compound ID	Molecular Structure	Pa	Pi	Biological Activity
1.	Lupinisolone C	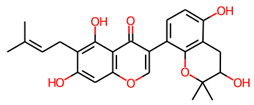	0.908	0.002	Histidine-kinase inhibitor
			0.866	0.003	Anticarcinogenic
			0.851	0.002	MMP9 expression inhibitor
			0.847	0.008	TP53 expression enhancer
			0.817	0.003	Antioxidant
2.	Gummadiol	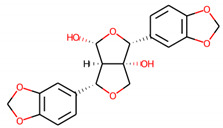	0.916	0.005	Antineoplastic
			0.901	0.011	Membrane integrity agonist
			0.695	0.018	Antidyskinetic
			0.679	0.030	TP53 expression enhancer
			0.644	0.005	Caspase 8 stimulant
3.	2,3-Dehydrokievitone	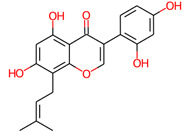	0.773	0.014	TP53 expression enhancer
			0.755	0.005	Histidine kinase inhibitor
			0.764	0.017	Antineoplastic
			0.746	0.011	Apoptosis agonist
			0.698	0.007	MMP9 expression inhibitor
4.	Tuberosin	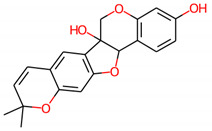	0.886	0.006	HIF1A expression inhibitor
			0.841	0.003	Chemopreventive
			0.800	0.002	NOS2 expression inhibitor
			0.792	0.013	Antineoplastic
			0.727	0.021	TP53 expression enhancer

## Data Availability

Not applicable.
